# "Is Cybermedicine Killing You?" - Cochrane Collaboration Needs to Restore Confidence

**DOI:** 10.2196/jmir.7.4.e37

**Published:** 2005-07-28

**Authors:** Myron L Pulier

I sat there agog reading your well-written, balanced, restrained, and devastating editorial on the "Cochrane disaster" [1]. I come away with the feeling that something is very wrong at Cochrane, and I'm extremely curious as to what it might be. Cochrane seems like such a good idea — so dignified, honorable, and professional — how could people get so sloppy and cavalier? If their false conclusions were of political value (defending a war, a contract, a questionable appointment) one would feel certain of having come across another outrageous conspiracy. But I can't imagine what cabal would substantially profit from finding that educating patients degrades their medical outcome. Was this just initial negligence, inattention, and then a sudden steamroller effect when a surprising finding reported by a bumbling research assistant seemed so novel and newsworthy that it attracted a misguided enthusiasm and loyalty that blinded all the participants to reality, with a perfunctory referee ritual proving inadequate to catch the errors?

I've been expressing skepticism about evidence-based medical practice at our departmental meetings but was gradually being won over by that approach, but now...whose evidence? Meta-analyzed by whom? I'm not competent to understand meta-analyses and sort of took them on faith. Fie on that.

I thought the editorial was very valuable and had some wise suggestions. I hope it will receive enough attention to lead to real changes in Cochrane procedures and in journals in general. One cannot assume that the chain of failures described in the editorial is unique. Certainly a diligent review of the Cochrane processes followed by appropriate candidly described fixes, perhaps as suggested by the editorial, are called for to restore confidence in what once seemed a reliable source of guidance for evidence-based clinical practice.

## References

[1] Eysenbach Gunther, Kummervold Per Egil (2005). "Is cybermedicine killing you?" - the story of a Cochrane disaster. J Med Internet Res.

